# Optimized and scalable synthesis of magnetic nanoparticles for RNA extraction in response to developing countries' needs in the detection and control of SARS-CoV-2

**DOI:** 10.1038/s41598-020-75798-9

**Published:** 2020-11-04

**Authors:** Julio C. Chacón-Torres, C. Reinoso, Daniela G. Navas-León, Sarah Briceño, Gema González

**Affiliations:** 1 School of Physical Sciences and Nanotechnology, Yachay Tech University, 100119 Urcuquí, Ecuador; 2 School of Chemical Sciences and Engineering, Yachay Tech University, 100119 Urcuquí, Ecuador

**Keywords:** Nanoparticles, Synthesis and processing

## Abstract

Ecuador is one of the most affected countries, with the coronavirus disease 2019 (COVID-19) infection, in Latin America derived from an ongoing economic crisis. One of the most important methods for COVID-19 detection is the use of techniques such as real time RT-PCR based on a previous extraction/purification of RNA procedure from nasopharyngeal cells using functionalized magnetic nanoparticles (MNP). This technique allows the processing of ~ 10,000 tests per day in private companies and around hundreds per day at local Universities guaranteeing to reach a wide range of the population. However, the main drawback of this method is the need for specialized MNP with a strong negative charge for the viral RNA extraction to detect the existence of the SARS-CoV-2 virus. Here we present a simplified low cost method to produce 10 g of nanoparticles in 100 mL of solution that was scaled to one litter by parallelizing the process 10 times in just two days and allowing for the possibility of making ~ 50,000 COVID-19 tests. This communication helps in reducing the cost of acquiring MNP for diverse biomolecular applications supporting developing country budgets constraints and chemical availability specially during the COVID-19 International Health Emergency.

## Introduction

The prevailing pandemic originated from the unusual coronavirus SARS-CoV-2 that causes the COVID-19 infectious disease has been striking the medical health system world wide due to its rapid propagation person-to-person^1–4^. A prompt and accurate detection method is crucial for the detection of COVID-19 in order to keep its proliferation under control. Recently the genome sequences of SARS-CoV-2 have been fully revealed and thus the use of techniques like real-time reverse transcription polymerase chain reaction (RT-PCR), has been widely used and developed as kits for the clinical diagnosis of COVID-19. Zhao et al*.* have shown the possibility of fabricating “simple magnetic nanoparticles” that can be further implemented into the RNA magnetic extraction method that will be used later as template in the real time RT-PCR for the clinical diagnosis of COVID-19^5^. However, the main drawback of this technique is the need for specialized and homogenous magnetic nanoparticles with a strong negative charge, so that the viral RNA extraction and purification process becomes faster and cleaner improving the number of clinical tests per day for COVID-19. Although, even if this technique can be implemented in every country, developing countries like Ecuador and many others in Latin America are struggling to implement it due to the relatively high prices of MNP in the international market and their import time frame. An example of this problem is confirmed by the city of Guayaquil in Ecuador, that has suffered extremely from the results of late detection and control along the explosive expansion of the novel coronavirus^6^.

In this communication, a 'simplified three step method' to produce large quantities of MNP for RNA extraction based on Zhao et al*.* synthesis^5^ is presented, optimized and scaled: a) Coating of magnetite nanoparticles (MNP) with APTES to form the amino-magnetic nanoparticles compound (NH_2_-MNP), b) Diacrylate-amine polymerization to obtain the Poly (amino-ester), c) Coating of amino-magnetic nanoparticles (NH_2_-MNP) with Poly (amino-ester) to form the final nanoparticle compound (Poly-NH_2_-MNP). This work brings a systematic characterization of the produced MNP along their fabrication process and it can be used as a benchmark in the correct synthesis of homogeneously MNP specialized for a high quality RNA extraction along their potential implementation into a real time RT-PCR for SARS-CoV-2 detection.

## Results and discussion

### Simplified preparation of magnetic nanoparticles

**Figure 1:**
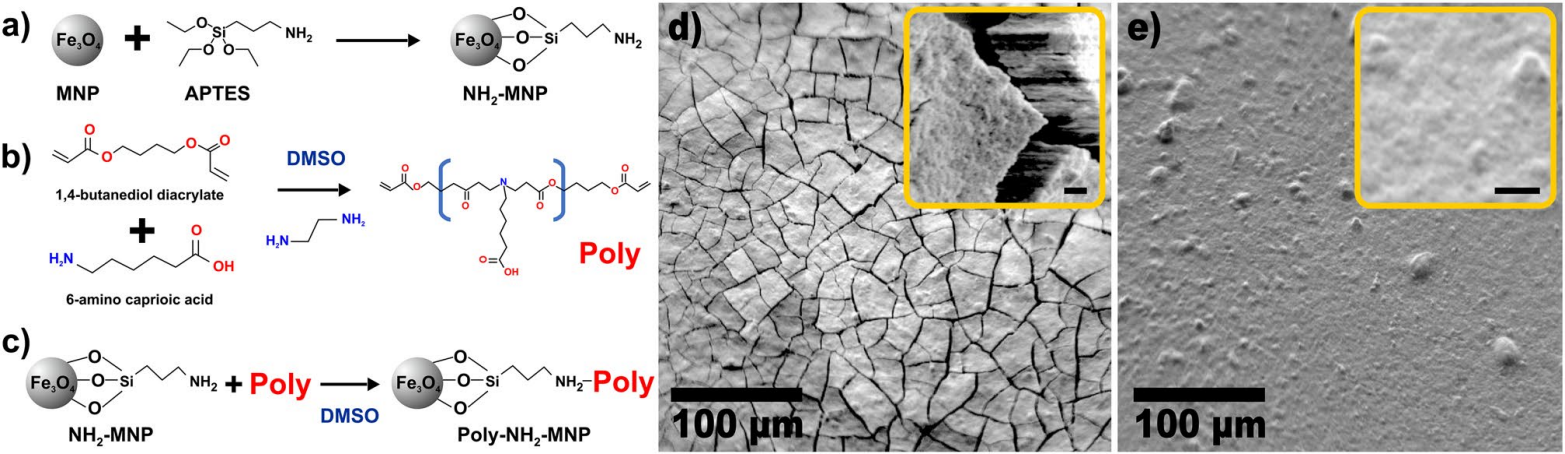
Schematic representation of the MNP synthesis and their resulting morphology. (**a**) Synthesis of amino-magnetic nanoparticles (NH_2_-MNP). (**b**) Poly (amino-ester) is synthesized by the combination of 1,4-butanediol diacrylate + 6-aminocaproic acid at DMSO solution via diacrylate-amine polymerization. (**c**) The final amino-magnetic nanoparticles coated with the Poly (amino-ester) material are synthesized by following a Michael addition methodology^8^ as introduced by Zhao et al.^5^ and represented as Poly-NH_2_-MNP in the following. (**d**) Magnetic nanoparticles dispersion dried on a SiO_2_ wafer and placed in ultra high vacuum conditions (UHV, ~ 10^-9^ mbar). The observed morphology of this sample reveals a rough compact surface derived from the intrinsic magnetic interaction between the nanoparticles covered with APTES. (**e**) Final nanostructured magnetic nanoparticles dispersion dried on a SiO_2_ wafer and placed in ultra high vacuum conditions (UHV, ~ 10^–9^ mbar). The observed morphology of this sample revealed a smooth continuous surface derived from the presence of the polymer on the MNP as active electronegative coating. Insets scale bar 10 µm.

The methodology for the preparation of magnetic nanoparticles coated with a negatively charged polymer (Poly) to be used in the extraction of RNA from the SARS-CoV-2 is represented in Fig. 1a–c. The magnetic nanoparticles were prepared using a simple and low-cost co-precipitation method followed by a hydrolysis process using 3-aminopropyl triethoxysilane (APTES) (Fig. 1a). In parallel, the poly (amino-ester) was prepared based on a modification of the protocol reported by Zhao et al*.*^5^ and Sunshine et al*.*^7^ by a diacrylate-amine polymerization using 1,4-butanediol diacrylate and 6-aminocaproic acid. (Fig. 1b). Finally, the amino-magnetic nanoparticles (NH_2_-MNP) were coated with the poly (amino-ester) material by following a Michael addition methodology^8^ in order to completely coat the magnetic nanoparticles with the polymer (Poly-NH_2_-MNPs) (Fig. 1c) introducing the desired negative charge required for the proper extraction of RNA. The synthesis protocol reported here was designed to produce 100 mL at (10% w/v) of Poly-NH_2_-MNP to fill the required amount of magnetic nanoparticles for their implementation into a real time RT-PCR for SARS-CoV-2 detection in Ecuador. In contrast to the experiment conducted by Zhao et al*.* where 40 g of polymer per gram of NH_2_-MNP were used^5^, we employed down to 0.2 mL of polymer (~ 0.1 g at 0.1% w/w) per gram of NH_2_-MNP. It is important to state at this point that our synthesis process did not include the use of tetraethyl orthosilicate (TEOS) and the magnetite nanoparticles were directly functionalized with (3-aminopropyltriethoxysilane –APTES–). On the other side, the main purpose of the coating is to have a negative charge on the surface of the nanoparticles. The latter optimization implies a considerable reduction in time and cost on the scaling up process. A 100 mL of Poly-NH_2_-MNP will permit the execution of about 5000 RNA extraction/purification procedures from nasopharyngeal cells that will be used as a template in the real-time RT-PCR tests for COVID-19 detection. However, the way that this epidemic has been developing around the world requires a more persuasive and robust method that could cover the real needs of developing countries as the amount of people to be tested is much higher every day. Thus, we scaled the synthesis method (Supplementary Fig. 2a) by parallelizing simultaneously each step from Fig. 1 being able to increase the production of MNP in one order of magnitude. We are able to obtain 1 L of Poly-NH_2_-MNP (10% w/v) in just two days in a basic laboratory. This result will allow the execution of about 50,000 real time RT-PCR tests for COVID-19 detection in Ecuador and become a potential solution for developing countries.

### Characterization of magnetic nanoparticles as prepared for the RNA extraction method

**Figure 2:**
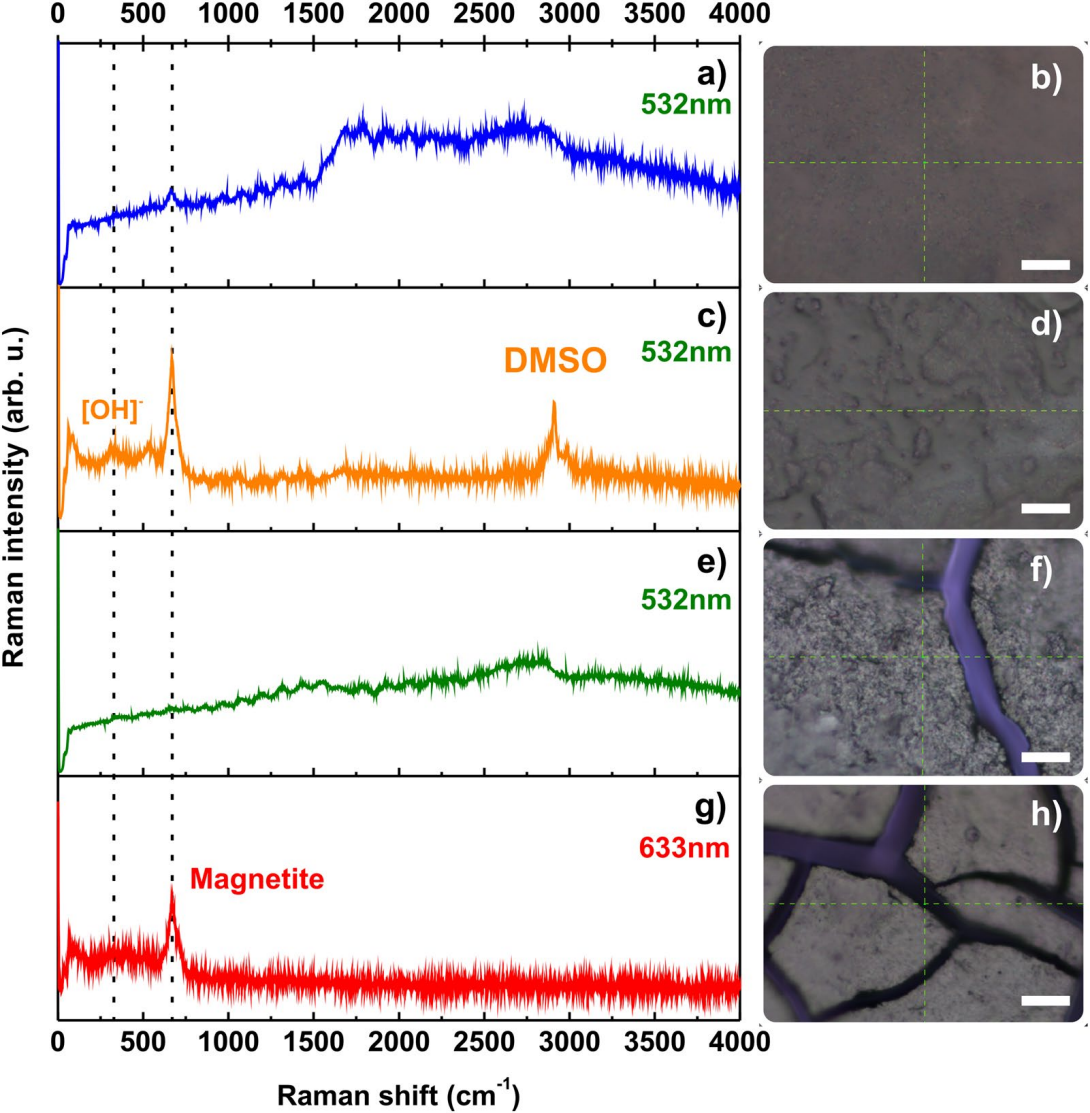
Raman spectrum of MNP measured at 532 nm excitation wavelength. Scale bars are of 100 µm. (**a**) Final nanostructured magnetic nanoparticles dispersion dried on a SiO_2_ wafer under ambient conditions. The high background observed is the result of the presence of the polymer coating (Poly) on the MNP. The characteristic 670 cm^−1^ peak from magnetite is still observable, which confirms an efficient coverage. (**b**) 100X Optical image of the final MNP revealing an homogenous smooth continuous surface as a result of an efficient coverage with Poly. (**c**) Magnetic nanoparticles dispersed in DMSO after being coated with APTES dried on a SiO_2_ wafer under ambient conditions. The strong 670 cm^−1^ peak confirms a high quality magnetite bound to DMSO through the presence of a doublet peak around 2915 cm^−1^ (C–H) symmetric stretch^10,11^. The 380 cm^−1^ peak revealed the [OH]– groups attached to magnetite according to reference^12^ and derived from the ethanol washing and drying procedure in air. (**d**) 100X Optical image of the DMSO dispersed MNP revealing an homogenous rough surface as a result of an efficient DMSO coverage/dispersion effective for a further Poly treatment. (**e**) APTES coated nanostructured magnetic nanoparticles dispersion dried on a SiO_2_ wafer under ambient conditions. The high background observed is the result of the presence of the APTES molecule that hinders the magnetite Raman response. (**f**) 100X Optical image of the NH_2_-MNP nanostructure revealing compact rough surface. No damage to the magnetite NP was observed after the measurements as a result of an effective APTES coating. (**g**) Initial MNP dispersion dried on a SiO_2_ wafer before coating. The characteristic peak of magnetite at 670 cm^−1^ confirms a high quality magnetite pristine material at 633 nm excitation. (**h**) 100X Optical image of the MNP surface revealing a rough non continuous surface.

A morphological analysis of the samples (Fig. 1d,e) was conducted using a scanning electron microscopy (SEM) based on a secondary electrons detector from a Versa Probe III X-ray photoelectron spectrometer. In Fig. 1d we confirm that our first treatment for dispersion of MNP was successful and our pristine magnetite nanoparticles were coated. The latter is an important and crucial property of the synthesis of MNP for RNA extraction as the succes in further coatings processes (Fig. 1a–c) is determined by an effective initial dispersion and coating of the MNP. The evident cracks along the surface of the dried dispersion of MNP on the SiO_2_ wafer (Fig. 2d) confirm the presence of a rough compact surface derived from the intrinsic magnetic interaction between the nanoparticles covered with APTES that may shrink during the drying process. The latter was double checked by depositing a drop of the MNP independently on an Al and Ti wafers. We noticed the presence of MNP clusters (see Supplementary Fig. 4a,b and e,f accordingly). In contrast, for the final end of this synthesis process, one desires to have a continuous and heterogeneous polymer/nanoparticle system that allows the correct performance along the RNA extraction technique. In Fig. 1e we corroborate this fact by a noticeable smooth and continuous surface derived from the presence of the poly (amino-ester) coating within the NH_2_-MNP. The latter was verified by the deposition of a drop of the Poly-NH_2_-MNP independently on an Al and Ti wafers. The presence of a continuous layer formed on the metal was evident to the extent of the resolution of the equipment as it can be observed in the Supplementary Fig. 4c,d and g,h respectively. We can still identify small agglomeration sites before and after the coating with Poly that are the result of the magnetic interaction between the nanoparticles during the drying process over the substrate. Nevertheless, it is important to highlight the fact that the coating on the magnetic nanoparticles with APTES and the polymer could reduce their intrinsic magnetic properties of the nanoparticles^9^. However, as it will be shown in the following, the precise characterization of the coating process will bring two main pieces of information: (1) the correct coating formation is as a good indicator of the APTES and Poly MNP’s functionalization required for the RNA binding, and (2) the coating should be enough to bring enough negative charge to the nanoparticle, but not too large so that the magnetic extraction procedure for RT-PCR works properly (see Supplementary Fig. 5).

In order to prove the efficiency of the coating process described in Fig. 1a–c, we employed Raman spectroscopy as a fast and precise characterization tool for nanomaterials. This method is well known in the characterization of magnetic nanoparticles^12,13^ and their interaction with organic solvents, polymers, and molecules^10,14^. In Fig. 2, the evolution of the Raman spectrum from the MNP is presented. We can trace the scheme in Fig. 1 with respect to the obtained Raman spectra as follows: i) As a first step, the MNP have to be synthesized and dispersed homogeneously in isopropanol solution. We found the characteristic peak from magnetite (Fe_3_O_4_)^12,15^ at 670 cm^−1^ using a 633 nm laser excitation and low laser power < 1 mW (Fig. 2g—red). We also featured the presence of a peak at ~ 380 cm^−1^ resulting from the [OH]^-^ groups linked to the surface of magnetite nanoparticles (Fig. 2c—orange) derived from the ammonium hydroxide and ethanol used during the synthesis of MNP nanoparticles in addition to the drying process performed on the SiO_2_ substrate under ambient conditions that open the possibility of humidity absorption along with the Raman analyses. ii) The second step of the synthesis requires a special coating with APTES molecules that eclipse the magnetite Raman response by a broad background as observed in Fig. 2e. iii) In a third step the APTES treated MNP (NH_2_-MNP) are dissolved and washed with DMSO enhancing the magnetite Raman response at 670 cm^−1^ due to a slight decoating of the initial APTES shell within the additional emergence of a double peak around 3000 cm^−1^ characteristic of DMSO. iv) Finally, the nanostructured NH_2_-MNP is binded to a polymer (amino-ester) that will serve as a negative charge carrier for the optimal RNA extraction methodology. Our Raman measurement of this final sample (Poly-NH_2_-MNP) shows again a broad background characteristic of a fluorescent polymer at 532 nm in addition to a perceptible signal of magnetite at 670 cm^−1^. During each Raman measurement, optical images of the samples were taken (Fig. 2b,d,f,h) to ensure no degradation of the sample during the measurement, and a surface morphological confirmation when compared to Fig. 2d,e.

**Figure 3:**
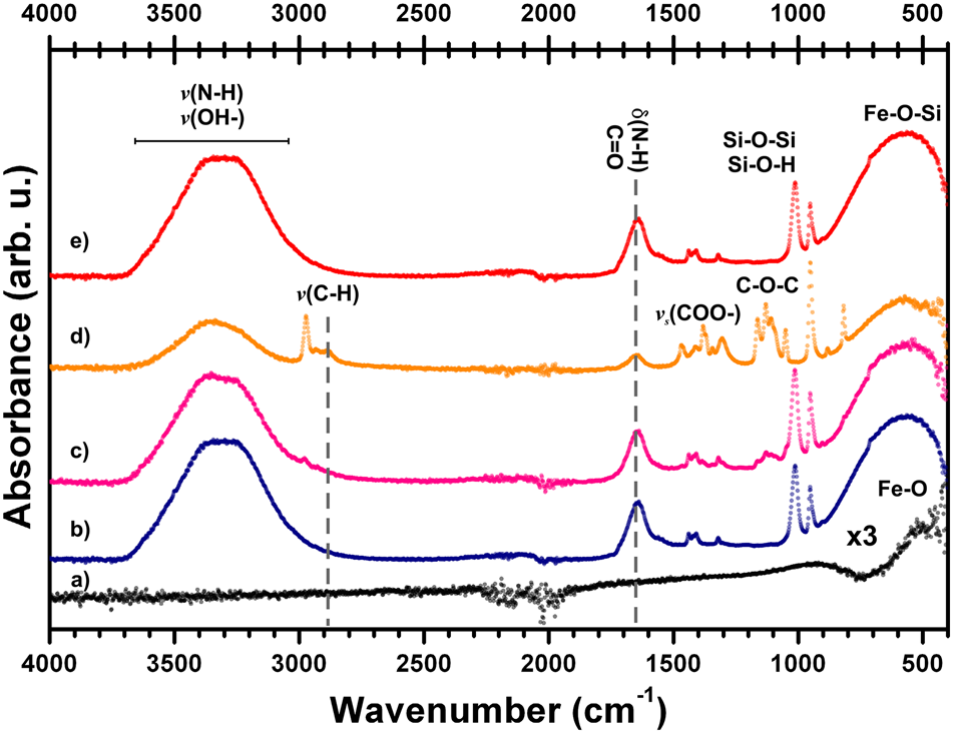
Infrared spectrum of MNP. (**a**) Magnetic nanoparticles (MNP), (**b**) amino-magnetic nanoparticles (NH_2_-MNP) (**c**) amino-magnetic nanoparticles (NH_2_-MNP) in DMSO, (**d**) Poly (amino-ester) and (**e**) amino-magnetic nanoparticles coated with Poly (amino-ester) (Poly-NH2-MNP).

**Figure 4:**
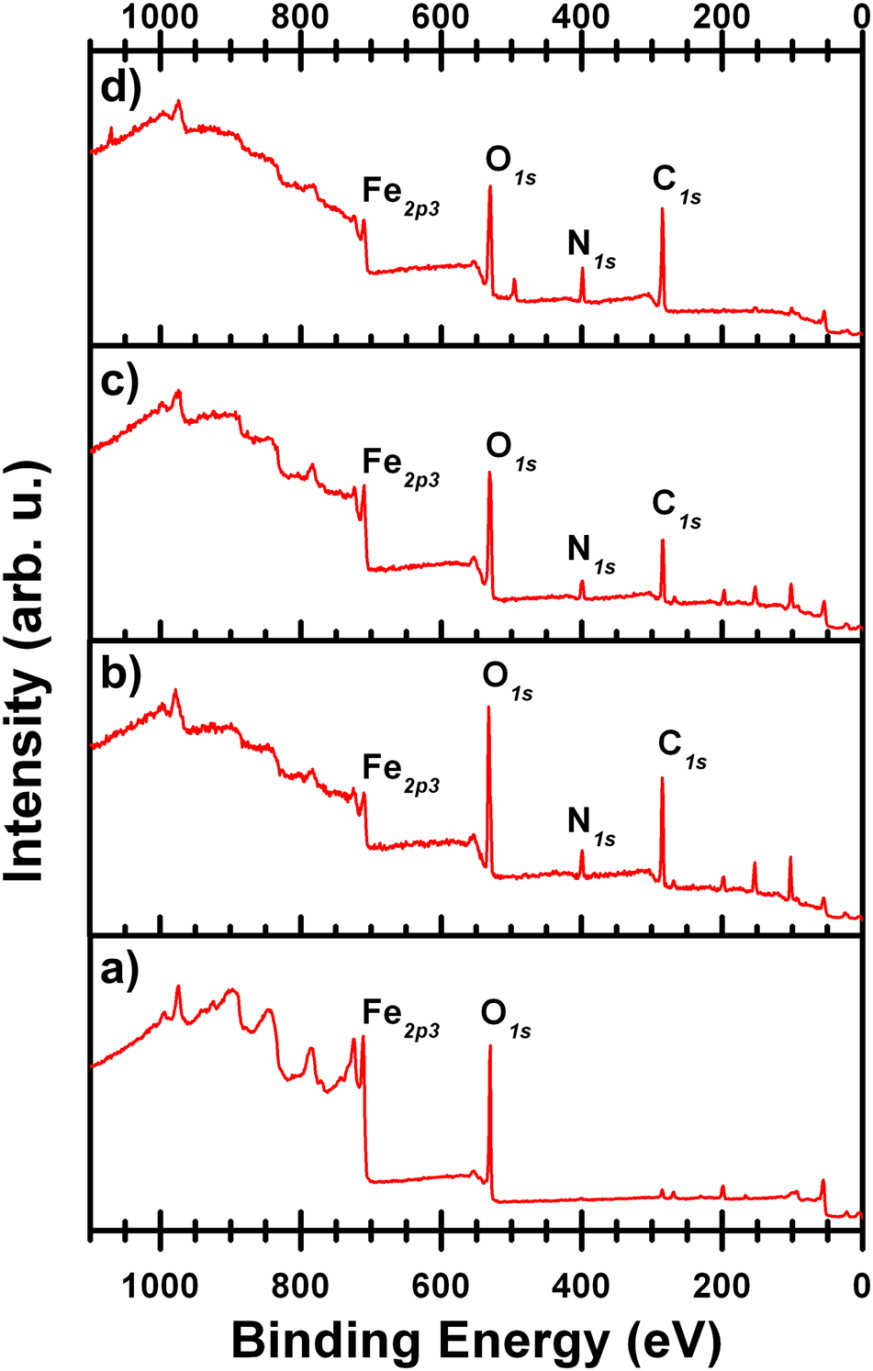
X-ray photoelectron spectra of MNP. (**a**) magnetic nanoparticles (MNP), (**b**) amino-magnetic nanoparticles (NH_2_-MNP) (**c**) amino-magnetic nanoparticles (NH_2_-MNP) in DMSO, (**d**) Poly (amino-ester) coated amino-magnetic nanoparticles (Poly-NH_2_-MNP).

In order to verify the presence of the final nanoparticle coating, that is a crucial step for the effectiveness of the MNP when used in the RNA extraction method, we used two accurate standar techniques: a) Infrared spectroscopy (Fig. 3), and b) X-ray photoelectron spectroscopy (XPS—Fig. 4).

In Fig. 3a the spectrum of magnetite nanoparticles is shown and confirmed by the presence of *v*(Fe–O) stretching vibration at ~ 572 cm^−1^^12,16^. The distinctive Fe–O–Si band is present around 580 cm^−1^ ^17^ and overlaps with the Fe–O vibration band, however it can be observed that the band in this region considerably increases the intensity, supporting the adsorption of APTES molecules on the magnetite surface (Fig. 3a to c) as expected from the first step in Fig. 1a.

The particles coated with APTES (NH_2_-MNP) show the amino groups in the region from 3200–3400 cm^−1^ corresponding to a N–H stretching and at 1630 cm^−1^ for the N–H bending vibrations. The characteristic Si–O–Si and Si–OH vibrations are present in the region from 1050–1100 cm^−1^.

The infrared measurement of the prepared reference negatively charged polymer coating (Poly) shows a broad band vibration of N–H and –OH groups in the range of 3700–3000 cm^−1^. The bands at 2860–2920 cm^−1^ assigned to C–H stretching symmetric and asymmetric vibrations of the ethylenediamine and the amide bands at 1576 cm^−1^. The vibrations at 1682–1685, 1111 and 1711 cm^−1^ correspond to the acrylate group (n C=O), the C–O–C and the carbonyl C=O stretching group respectively, while the 1313–1346 cm^−1^ component is attributed to the carboxylic acid stretching mode v_s_(COO–).^18^.

The infrared spectrum confirms the efficient coating of MNP with Poly. According to Zhao et al*.*^5^ this particular negatively charged coating is essential for the optimal extraction and purification of the RNA sample that will improve the performance of the real time RT-PCR analysis process in the detection of SARS-CoV-2^5^.

The final chemical surface characterization for the MNP was conducted using XPS spectroscopy. As it is shown in Fig. 4, the binding energy comparative survey analysis exhibit clear features among the samples:

1. The iron analysis in XPS spectroscopy is a tool to confirm the presence of magnetite (Fe_3_O_4_) nanoparticles. Our initial material (Fig. 4a) revealed the strongest peak from the Fe2p3 core level with a 24.8% atomic concentration. By performing a high resolution XPS analysis in the region from 705 to 730 eV, we confirmed the presence of magnetite by a doublet peak corresponding to the Fe2p_1/2_ and Fe2p_3/2_^15^ components (Supplementary Fig. 3a). After the first coating process (Fig. 4b), we observed an attenuation down to 3.2 (atm%) of the Fe peak derived from the presence of NH_4_OH deposited on the MNP’s surface. After washing the MNP with DMSO, the signal intensity of Fe2p3 raised to 7.7 (atm%) in Fig. 4c as the DMSO washed out the excess of the non-attached NH_4_OH coating material. In the final step (Fig. 4d), the nanoparticles were coated with a polymer (3-Aminopropyl) and thus the signal of magnetite was strongly reduced to less than 0.1 (atm%). In the end, we confirmed that the final coating created on the MNP is of around 9 nm, which is the limit for photon penetration in the sample coming from the XPS surface technique.
2. Regarding the N1s core level (~ 400 eV) analysis, we found this peak to be absent (as expected) in the pristine MNP sample in Fig. 4a due to the lack of amino groups. After the first coating with NH_4_OH the nitrogen concentration was increased to 8.4% (Fig. 4b). Subsequently, the excess of NH_4_OH molecules was washed by DMSO (Fig. 4c). We noticed then a reduction in the nitrogen atomic concentration to 7.5% as is shown in Table 1. We attribute this effect, as observed in the infrared analysis, to the removal of some NH_4_ groups from the first coating surface of the nanoparticles. In the final step (Fig. 4d), when the MNP are being coated with 3-Aminopropyl (Poly-NH_2_-MNP), the nitrogen concentration is raised to 10.3 atomic% (Table 1). The latter nitrogen development is a clear signature of the successful coating process essential to have magnetic nanoparticles covered with a negative charged polymer for the RNA extraction. The detailed development of the nitrogen functional groups formation on the MNP can be observed in the Supplementary Information in a close region from 398 to 405 eV (Supplementary Fig. 3) showing the characteristic peaks corresponding to NH_4_OH and NH_2_ molecular elements present in the sample^19,20^.

**Table 1:** Atomic percentages at each coating stage process evaluated from the X-ray photoelectron spectra.

(Atomic%)	MNP	NH_2_-MNP	NH_2_-MNP in DMSO	Poly-NH_2_-MNP
O1s (%)	60.4	33.9	42.4	32.2
Fe2p3 (%)	24.8	3.2	7.7	< 0.1
C1s (%)	10.4	41.7	32.4	53.5
N1s (%)	–	8.4	7.5	10.3
Si2p (%)	–	11	8.7	1.3
Cl2p (%)	4.4	1.8	1.3	0.3
Na1s (%)	–	–	–	2.4
3. The carbon C1s signal in the pristine MNP (Fig. 4a) is minimum (~ 10% Table 1) denoting the clean precursors that mostly contain magnetite nanoparticles. The C1s core level appears at (284.8 eV) after the first coating deposition with 41.7 (atm%) in Fig. 4b and Table 1 correspondingly followed by a reduction (32.4%) due to the DMSO processing. At the final part of the process, the C1s signal increased in agreement to the 3-Aminopropyl addition as the last coating (Fig. 4d) having a 53.5 (atm %) on the sample.
4. The O1s feature at (532 eV) for MNP (Fig. 4a) has an atomic percentage of 60.4 with a final value of 32.2% at the final step (see Table 1) confirming an intensity reduction due the signal attenuation of the Fe_3_O_4_, leaving just the oxygen contribution from the 3-Aminopropyl coating. Traces of Cl, Si and Na are present at (~ 196 eV, 101 eV and 1072 eV correspondingly) due the composition of the precursors used in the synthesis and in each consecutive coating process as it is shown in Table 1, where atomic chemical elemental composition for each step is exhibited.

### Real time RT-PCR test results using RNA extracted with our synthesized Poly-NH_2_-MNP magnetic nanoparticles

The RNA extraction process is the first and most important step in the procedure to detect the SARS-CoV-2 virus, the MNP extraction method allows for an improved purification and quality factor of RNA in less time raising the number of clinical tests per day for COVID-19.

The methodology for real-time RT-PCR begins by transforming RNA into DNA by reverse transcription, then the DNA chain is opened to verify if the virus genes (RdRP, E and N) are present with complementary primers to those genes^21^. Attached to the ends of the primers are two molecules: a fluorophore and a quencher, which emit light when they are separated, so if both are close in the same primer there will be no light emitted^22^.

Since the genetic material of the virus is very small (29,891 nucleotides in size), millions of copies are needed to make it detectable^23^. Thus, it is essential to replicate it in a reaction of several cycles in which the primer disintegrates in such a way that the fluorophore keeps far away from the quencher and emits fluorescence^24^.

**Figure 5:**
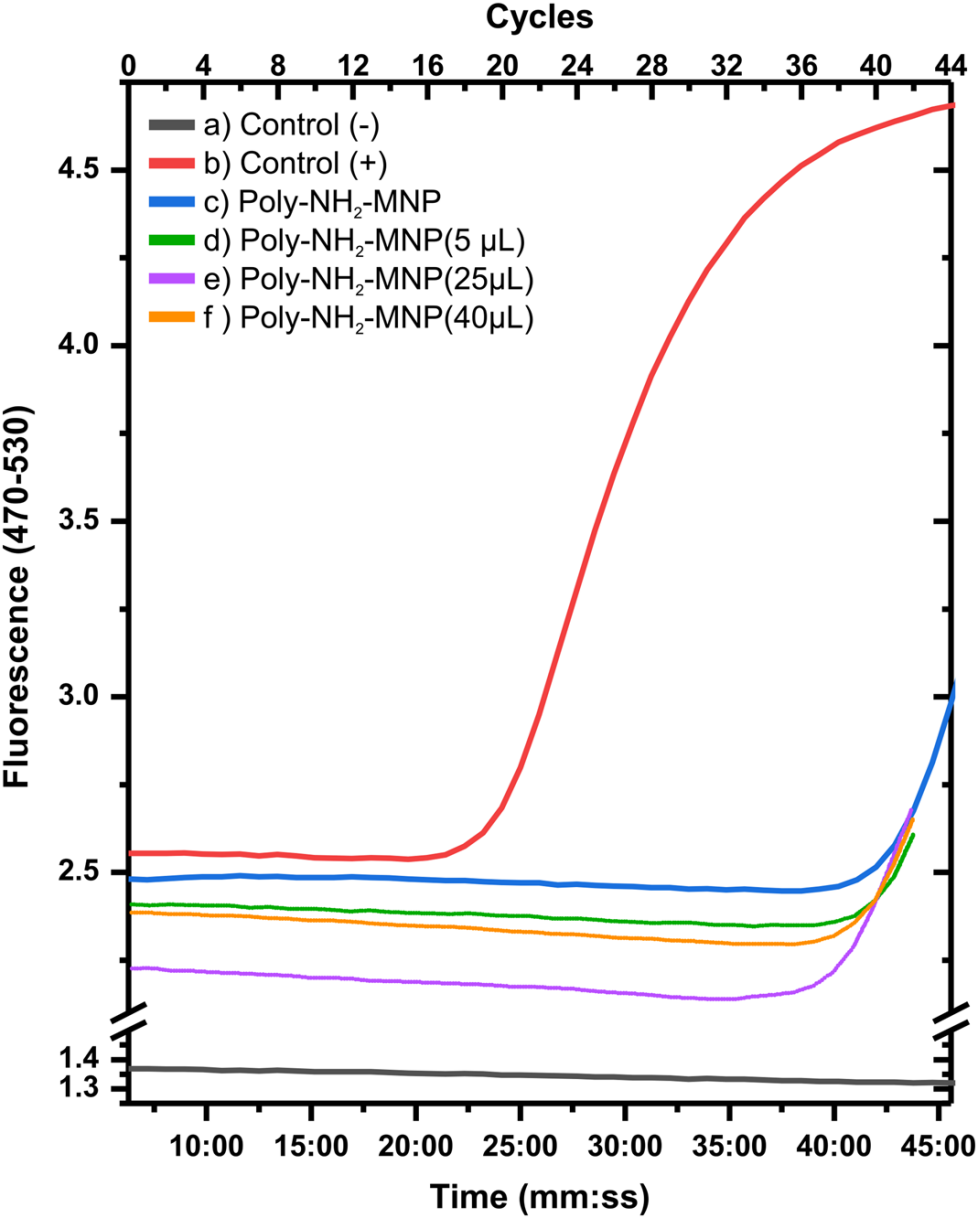
Real Time RT-PCR Amplification. After ~ 38 min (cycle 35 approx.) we observed an increase in the fluorescence when using Poly-NH_2_-MNP magnetic nanoparticles, being. (**a**) Negative control, qRT-PCR mix was employed with just 8 µL of ultrapure. (**b**) Positive control, 8 µL of viral RNA from a commercial extraction kit were placed together with the qRT-PCR mixture. (**c**) Positive sample experiment implementing 10 µL of our Poly-NH_2_-MNP magnetic nanoparticles instead of the ones from the qRT-PCR mixture coming in the commercial extraction kit. (**d**) The same extraction procedure was carried out as in (**c**) varying the amount of Poly-NH_2_-MNP magnetic nanoparticles to 5 µL. Finally, the volume of magnetic nanoparticles (Poly-NH_2_-MNP) employed in the qRT-PCR mixture was increased to 25 µL in (**e**) and 40 µL (**f**) accordingly. The observed amplification behavior remains relatively constant and independent of the Poly-NH_2_-MNP magnetic nanoparticles concentration which highlights their performance.

In Fig. 5 we present the results from the real time RT-PCR amplification analysis conducted by the private company InnovativeHealth LATAM in Quito-Ecuador. The results here revealed an increase in the fluorescence quantified in arbitrary fluorescence units approximately from the minute 38th (35th cycle) using a positive control sample, which establishes that the SARS-CoV-2 virus was present and its RNA was correctly extracted by using our Poly-NH_2_-MNP magnetic nanoparticles (Fig. 5c). Our real time RT-PCR amplification study proved that the RNA extraction method followed by the company using our synthesized Poly-NH_2_-MNP magnetic nanoparticles (see methods section) is functional but not as effective when compared to the processed data from RNA samples extracted using commercial MNP with the same positive control sample (Fig. 5a). We vary the concentration of nanoparticles in order to reveal the effect of this variable along the polymerase chain reaction and observed no substantial difference in the time of the fluorescence amplification (Fig. 5d–f). The time of the analysis for these three particular samples was stopped before the positive and negative control, as well as in the Poly-NH_2_-MNP magnetic nanoparticles (Fig. 5a–c). The latter confirms no quenching of the RT-PCR signal by increasing the concentration of nanoparticles in the solution and a constant reliable and accurate extraction behavior of our MNP that is of high importance when conducting thousands of tests per day. At this point our synthesis process did not include the use of tetraethyl orthosilicate (TEOS) and magnetite nanoparticles were directly functionalized with (3-aminopropyl) triethoxysilane (APTES) with a reduced concentration of the polymer of ~ 0.2 mL for each gram of NH_2_-MNP when compared to the experiment conducted by Zhao et. al. (40 g of polymer per gram of NH_2_-MNP)^5^. We consider it necessary to conduct further studies varying the type, size and coating of the magnetic nanoparticles in order to look for an optimal material for a greater qRT-PCR amplification response even if we already revealed a favorable response starting from 10 µL of magnetic nanoparticles.

## Conclusions

In summary, we reported a comprehensive study on the synthesis of functionalized coated MNP with a negatively charged polymer for viral RNA extraction and purification that could be implemented in the detection of the SARS-CoV-2 virus on a large scale. This work set a systematic characterization protocol in the synthesis of MNP that can be used as a benchmark for the correct fabrication of MNP specialized for high quality RNA extraction method before their potential implementation into a real time RT-PCR. The synthesis protocol reported here serves for the production of 100 mL at (10% w/v) of Poly-NH_2_-MNP that can be extended to 1L of Poly-NH_2_-MNP (10% w/v) in just two days in a basic laboratory (Supplementary Fig. 2) by parallelizing the synthesis process allowing for the execution of about 50,000 real time RT-PCR tests for COVID-19 detection. Our final 1L Poly-NH_2_-MNP (10% w/v) was sent to be tested as part of a real time RT-PCR analyses in a private company aiming for the optimal extraction of RNA to detect SARS-CoV-2 virus. By real time RT-PCR amplification analysis conducted by a private company in Ecuador we confirmed an increase in the fluorescence quantified in arbitrary fluorescence units at ~ 38 min., which confirms: a) the presence of the SARS-CoV-2 virus in the sample, and b) a correct RNA was extracted by using our Poly-NH_2_-MNP magnetic nanoparticles. With this work we (Supplementary Fig. 6) provided a solution to a national Latinamerican problem along the detection and control of the explosive expansion of the novel coronavirus despite the country budget constraints and chemical and laboratory facility availability during this declared International Public Health Emergency.

## Methods

### Infrared spectroscopy

The infrared analysis of the samples was done using an Agilent Technologies spectrometer Cary 360 with a diamond attenuated total reflectance (ATR) accessory and resolution of 4 cm^−1^. For each spectrum 32 scans acquisitions were compiled in a range between 400 and 4000 cm^−1^.

### Raman spectroscopy

The spectroscopic Raman detection was carried out under ambient conditions using a HORIBA LabRam spectrometer with a 514 nm and 633 nm excitation wavelengths at ~ 0.65 mW and 1.5 mW between − 50 and 4000 cm^−1^. To avoid laser-induced damage on the samples the laser power was kept below 1 mW. Raman measurements were carried out using a micro-Raman setup with a 100X short distance objective in backscattering geometry. A charge-coupled device is used to detect the signal after analyzing the signal via a monochromator similar to previous Raman spectroscopy studies we have conducted^14^. The spectrometer was calibrated in frequency with the Rayleigh peak to be set at 0 cm^−1^.

### X-ray photoelectron spectroscopy

The surface chemistry characterization was recorded by using X-Ray Photoelectron Spectroscopy (XPS) PHI VersaProbe III (Physical-Electronics) equipped with a 180 hemispherical electron energy analyzer and excited by a monochromatized Al-K_α_ source with an energy 1486.6 eV. Its energy analyzer operates in the pass energy mode at 280 eV for Survey and 55 eV for high resolution. The analysis spot had a diameter of 50 μm and 45° detection angle relative to the substrate surface. Atomic concentration for each element was calculated using Mutipak Version 9.8.0.19 (Ulvac-phi, Inc.) this software uses the peak intensity (peak area) in units of counts per second taking into account the specific relativity sensitivity factor for each element^25,26^. Background subtraction was not necessary due the high intensity peaks and non important energy losses prior to emission from the sample.

### RNA extraction and real time RT-PCR

To evaluate the effectiveness of the Poly-NH_2_-MNP, a commercial IVD kit was used with already standardized reagents and whose only differential variables were the Poly-NH_2_-MNP in different concentrations and in different extraction procedures.

The steps of the kit extraction protocol were followed (see Table 2) and 5, 10, 25 and 40 µL (microliters) of Poly-NH_2_-MNP were added to our nasopharyngeal swab samples along with a positive control of a known sample with the presence of the SARS-CoV-2 virus.

**Table 2:** Thermocycling qRT-PCR procedure established from a commercial IVD kit.

Step	Temperature (°C)	Time (min)	Cycles
Retro transcription	50	20	1
Denaturation	95	15	1
Denaturation 2	94	0:15	40
Amplification	58	0:30	
Cooling	40	0:30	

After obtaining the genetic material, 8 µL of extracted RNA (stored between 2 and 8 °C) was used to start the PCR with a final volume of 20 µL.

### Chemicals and solvents

Iron (III) chloride hexahydrate, Iron (II) chloride tetrahydrate, ammonium hydroxide, (3-Aminopropyl) triethoxysilane (APTES), and dimethyl sulfoxide (DMSO), isopropanol, ethylenediamine and ethanol, 1,4-butanediol diacrylate and 6-amino caproic acid were bought from Sigma-Aldrich Co. and used as received (99.99% purity).

### Synthesis of magnetic nanoparticles (MNP)

Magnetic nanoparticles (MNP) were prepared using a simple and low-cost co-precipitation method (Fig. 1a)^27^. 6.5 g of Iron (III) chloride hexahydrate and 3.5 g of Iron (II) chloride tetrahydrate were mixed in 100 mL of distilled water at 50 °C, and 20 mL of ammonium hydroxide was added into the mixture with vigorous stirring under a nitrogen atmosphere. A rapid change of solution colour was observed from orange to black, indicating the formation of MNP. The solution mixture was continuously stirred for another 30 min, and the resulting black products (MNP) were collected with a magnet and dispersed into ethanol after washing several times with deionized water and ethanol as in reference^27^.

### Synthesis of amino-magnetic nanoparticles (NH_2_-MNP)

Subsequently, the prepared MNPs were dispersed into 50 mL isopropanol, and 2 mL of APTES was dropwise mixed with MNPs solution (Fig. 1a)^28^. The mixture was incubated under continuous sonication for 3 h at room temperature, followed by the collection of amino-modified MNPs (NH_2_-MNP) with a magnet and washing with distilled water and ethanol to remove free APTES. The final prepared NH2-MNP was preserved in ethanol and the loading of the –NH_2_ group on the surface was verified by infrared spectroscopy (Fig. 3) as donde in reference^28^.

### Synthesis of polymer coated amino-magnetic nanoparticles (Poly-NH_2_-MNP)

Poly-NH_2_-MNP synthesis is adapted based on Zhao et al*.* protocol^5^. NH_2_-MNP were dispersed in 25 mL of 50% (v/v) DMSO aqueous solution and then mixed with 2.5 mL of Poly at 0.2 mL for each 10 g of NH_2_-MNP (Fig. 1c). Subsequently, 2.5 mL of NaOH solution (1 M) was introduced to the mixture, followed by vigorously stirring for 4 h at room temperature in a dark place, followed by the collection of Poly-NH_2_-MNP with a magnet and washing it with 50% (v/v) DMSO for several times. The obtained polymer coated MNP (Poly-NH_2_-MNP) was stored in distilled water at 4 °C in a dark place as explained in reference^5^. The loading of a carbonyl group of Poly (amino-ester) on the surface was verified by infrared spectroscopy, X-ray photoelectron spectroscopy, Raman spectroscopy and SEM techniques (Figs. 1, 2, 3 and 4).

## Supplementary information

Supplementary Information.

## Data Availability

The datasets generated during and/or analysed during the current study are available in the openICPSR repository, https://www.openicpsr.org/openicpsr/project/120310/version/V1/view/.

## References

[1] Paules CI, Marston HD, Fauci AS (2020). Coronavirus infections—more than just the common cold. JAMA.

[2] Li Q (2020). Early transmission dynamics in Wuhan, China, of novel coronavirus-infected pneumonia. N. Engl. J. Med..

[3] Wang D (2020). Clinical characteristics of 138 hospitalized patients with 2019 novel coronavirus-infected pneumonia in Wuhan, China. JAMA.

[4] Bai Y (2020). Presumed asymptomatic carrier transmission of COVID-19. JAMA.

[5] Zhao, Z. *et al.* A simple magnetic nanoparticles-based viral RNA extraction method for efficient detection of SARS-CoV-2. *bioRxiv* 2020.02.22.961268 (2020). 10.1101/2020.02.22.961268.

[6] Cdh, C. P. P. la D. de L. D. H. *et al.* Intervención humanitaria de la ONU para atender la gravedad de la crisis en Guayaquil por el COVID-19. (2020).

[7] Sunshine J (2009). Small-molecule end-groups of linear polymer determine cell-type gene-delivery efficacy. Adv. Mater..

[8] Payra S, Saha A, Banerjee S (2016). On-water magnetic NiFe2O4 nanoparticle-catalyzed Michael additions of active methylene compounds, aromatic/aliphatic amines, alcohols and thiols to conjugated alkenes. RSC Adv..

[9] Yamaura M (2004). Preparation and characterization of (3-aminopropyl)triethoxysilane-coated magnetite nanoparticles. J. Magn. Magn. Mater..

[10] Selvarajan A (1966). Raman spectrum of dimethyl sulfoxide (DMSO) and the influence of solvents. Proc. Indian Acad. Sci. Sect. A.

[11] Schuster JJ, Will S, Leipertz A, Braeuer A (2014). Deconvolution of Raman spectra for the quantification of ternary high-pressure phase equilibria composed of carbon dioxide, water and organic solvent: extraction of phase equilibrium data of ternary systems. J. Raman Spectrosc..

[12] Slavov L (2010). Raman spectroscopy investigation of magnetite nanoparticles in ferrofluids. J. Magn. Magn. Mater..

[13] Panta PC, Bergmann CP (2015). Raman spectroscopy of iron oxide of nanoparticles (Fe3O4). J. Mater. Sci. Eng..

[14] Vecera P (2017). Precise determination of graphene functionalization by in situ Raman spectroscopy. Nat. Commun..

[15] Minati L (2011). Application of factor analysis to XPS valence band of superparamagnetic iron oxide nanoparticles. Appl. Surf. Sci..

[16] Stoia M, Istratie R, Păcurariu C (2016). Investigation of magnetite nanoparticles stability in air by thermal analysis and FTIR spectroscopy. J. Therm. Anal. Calorim..

[17] Ahangaran F, Hassanzadeh A, Nouri S (2013). Surface modification of Fe3O4@SiO2 microsphere by silane coupling agent. Int. Nano Lett..

[18] Refat MS, El-Korashy SA, Kumar DN, Ahmed AS (2008). FTIR, magnetic, 1H NMR spectral and thermal studies of some chelates of caproic acid: inhibitory effect on different kinds of bacteria. Spectrochim. Acta A Mol. Biomol. Spectrosc..

[19] Orlando F (2016). Synthesis of nitrogen-doped epitaxial graphene via plasma-assisted method: role of the graphene–substrate interaction. Surf. Sci..

[20] Ewels CP, Glerup M (2005). Nitrogen doping in carbon nanotubes. J. Nanosci. Nanotechnol..

[21] Corman, V. M. *et al.* Detection of 2019 novel coronavirus (2019-nCoV) by real-time RT-PCR. *Euro Surveill.* **25**, 23–30 (2020). 10.2807/1560-7917.ES.2020.25.3.2000045 PMC6988269 31992387

[22] Lee, M. A., Squirrell, D. J., Leslie, D. L. & Brown, T. Homogeneous fluorescent chemistries for real-time PCR. In *Real-time PCR. Current technology and applications*. (eds Logan, J. *et al*.). 23–43 (Caister Academic Press, Norfolk, UK, 2009).

[23] Chan JF-W (2020). Genomic characterization of the 2019 novel human-pathogenic coronavirus isolated from a patient with atypical pneumonia after visiting Wuhan. Emerg. Microbes Infect..

[24] Navarro E, Serrano-Heras G, Castaño MJ, Solera J (2015). Real-time PCR detection chemistry. Clin. Chim. Acta.

[25] Wagner CD (1981). Empirical atomic sensitivity factors for quantitative analysis by electron spectroscopy for chemical analysis. Surf. Interface Anal..

[26] Seah MP, Gilmore IS, Spencer SJ (2001). Quantitative XPS: I. Analysis of X-ray photoelectron intensities from elemental data in a digital photoelectron database. J. Electron Spectrosc. Relat. Phenom..

[27] Yazdani F, Seddigh M (2016). Magnetite nanoparticles synthesized by co-precipitation method: the effects of various iron anions on specifications. Mater. Chem. Phys..

[28] Bini RA, Marques RFC, Santos FJ, Chaker JA, Jafelicci M (2012). Synthesis and functionalization of magnetite nanoparticles with different amino-functional alkoxysilanes. J. Magn. Magn. Mater..

