# Supplementary material for: Optimized and scalable synthesis of magnetic nanoparticles for RNA extraction in response to developing countries' needs in the detection and control of SARS-CoV-2
Source: Sci Rep. 2020 Nov 4;10:19004. doi: 10.1038/s41598-020-75798-9 (PMC7642403; doi:10.1038/s41598-020-75798-9)
Supplement: Supplementary file 1 — Supplementary Information. [file 41598_2020_75798_MOESM1_ESM.docx]

**Optimized and scalable synthesis of magnetic nanoparticles for RNA extraction in response to developing countries' needs for the detection and control of SARS-CoV-2.**

*Julio C. Chacón-Torres^1*^, C. Reinoso^1^, Daniela G. Navas-Leon^2^, S. Briceño^1^ & G. González^1*^*

(1) Yachay Tech University, School of Physical Sciences and Nanotechnology, Urcuquı́ 100119, Ecuador.

(2) Yachay Tech University, School of Chemical Sciences and Engineering, Urcuquı́ 100119, Ecuador.

Tel.: (+593) (6) 2999130 ext. 2612
E-mail:jchacon@yachaytech.edu.ec
E-mail: ggonzalez@yachaytech.edu.ec


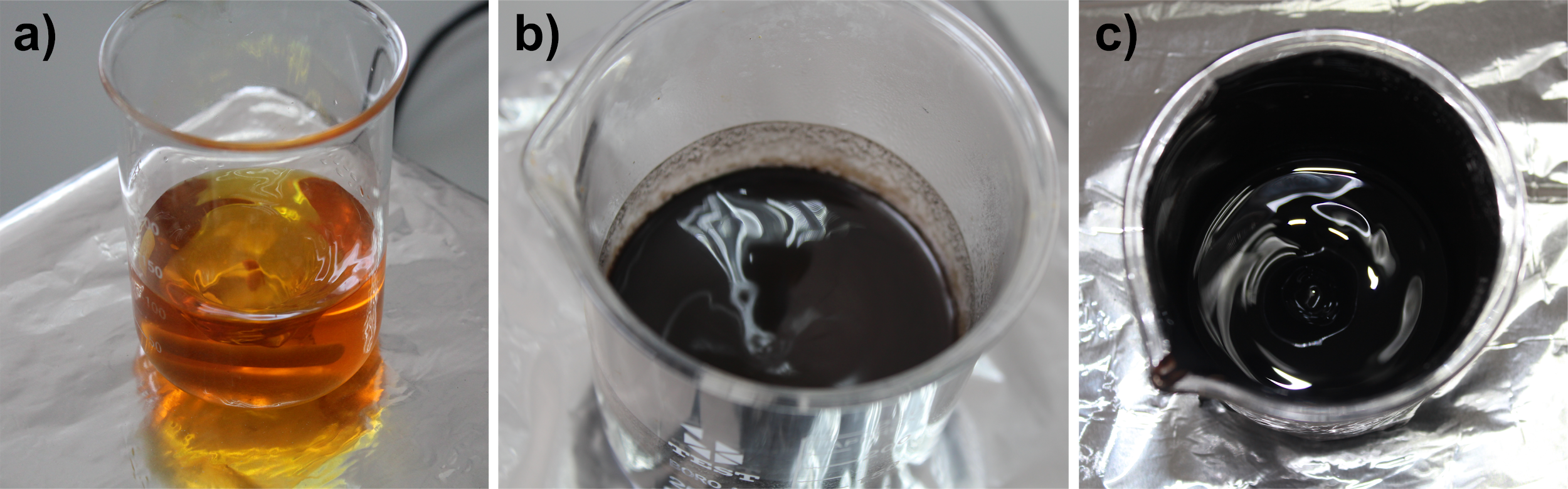


**Figure 1**: Changes in the solution color during the co-precipitation reaction: a) Nucleation process of Fe^3+^/Fe^2+^ b) Magnetic nanoparticles (MNP), c) Poly (amino-ester) coated amino-magnetic nanoparticles (Poly-NH_2_-MNP). During the synthesis, the reaction medium was heated and stirred at 50ºC and the nucleation process started Figure 1a manuscript (MS), when ammonium hydroxide was added into the mixture a rapid change of solution color was observed from orange to black (b) indicating the formation of MNPs. We can see that with the incorporation of the amine groups and the polymer (c) the sample maintains the dark black color without oxide.


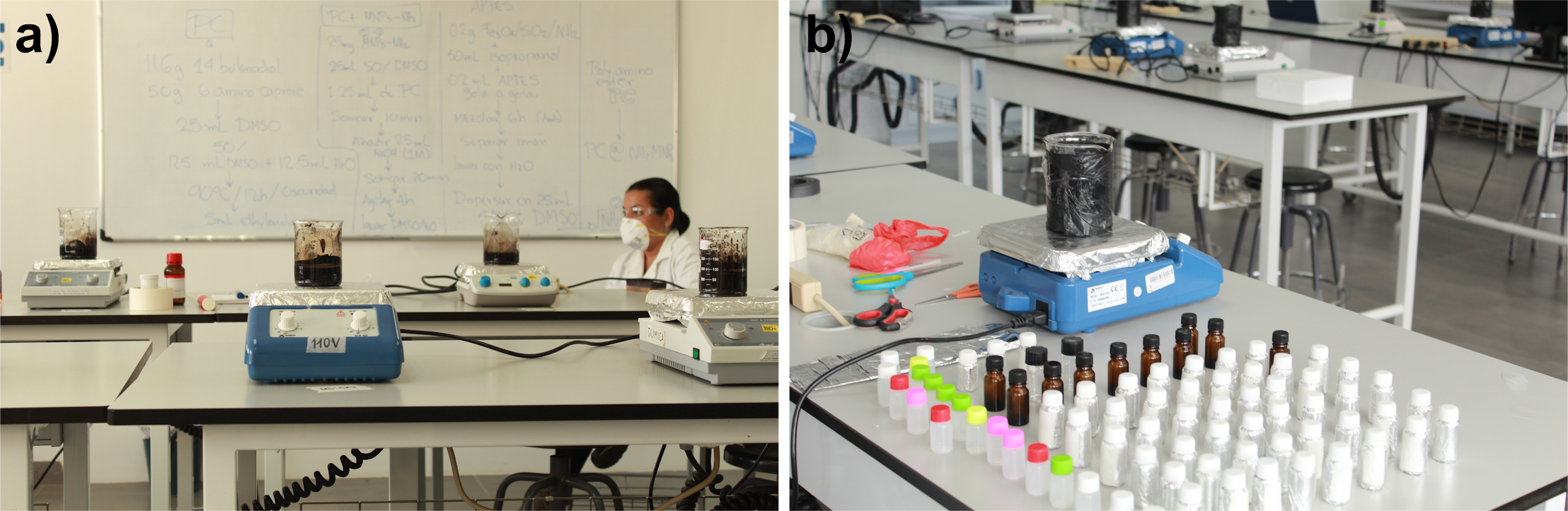


**Figure 2**: a) Scaled the synthesis method. Parallelization of the MNP production from Figure 1 MS to obtain 1 L of Poly-NH_2_-MNP (10 % w/v) in two days in a basic laboratory. b) Poly-NH_2_-MNP packing. The MNP were placed in vials of 10 mL and covered with aluminum foil against sunlight. Each vial serves for 50 ARN extraction tests along the real time RT-PCR analyses.


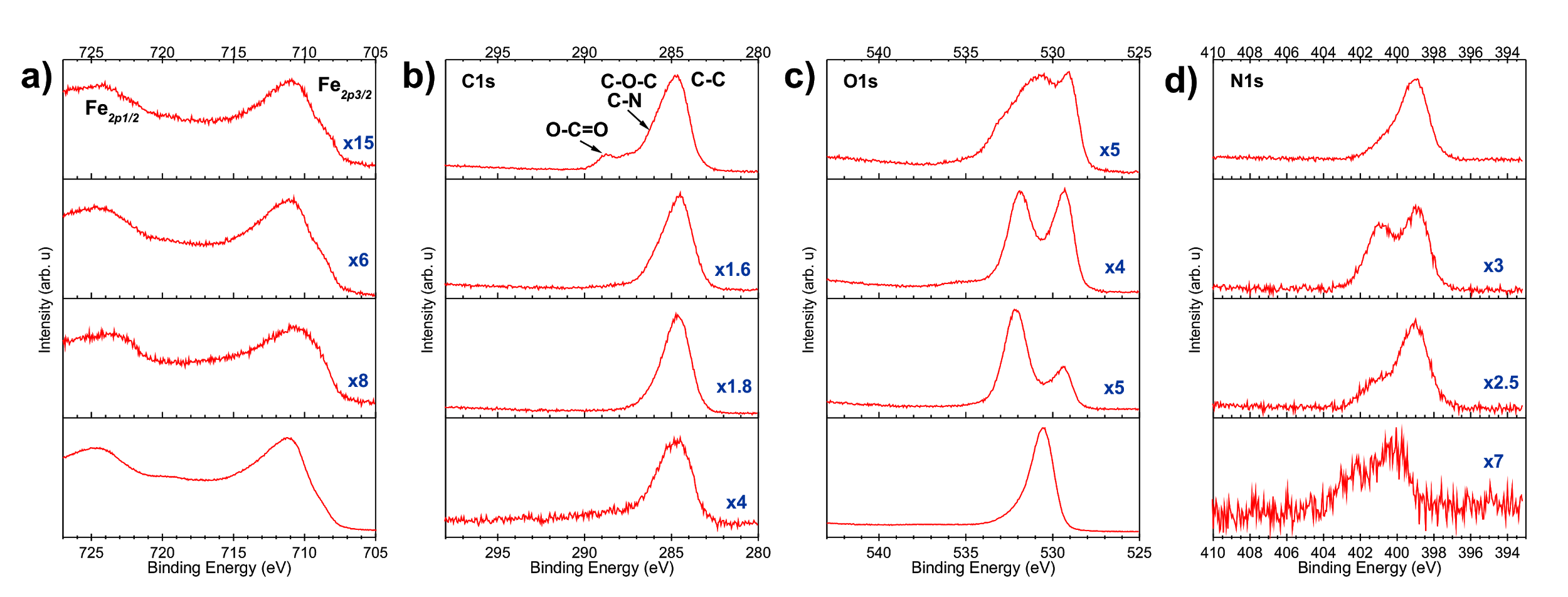


**Figure 3**: Individual High Resolution X-ray photoelectron spectra of iron Fe_2p1/2_, carbon C1s, oxygen O1s and nitrogen 1s core levels of elements present in MNP. The spectral data exhibit here correspond from bottom-up to: magnetic nanoparticles (MNP), amino-magnetic nanoparticles (NH_2_-MNP), amino-magnetic nanoparticles (NH_2_-MNP) in DMSO, and top Poly (amino-ester) coated amino-magnetic nanoparticles (Poly-NH_2_-MNP). a) Fe2p, b) C1s, c) O1s, and N1s core level analyses.


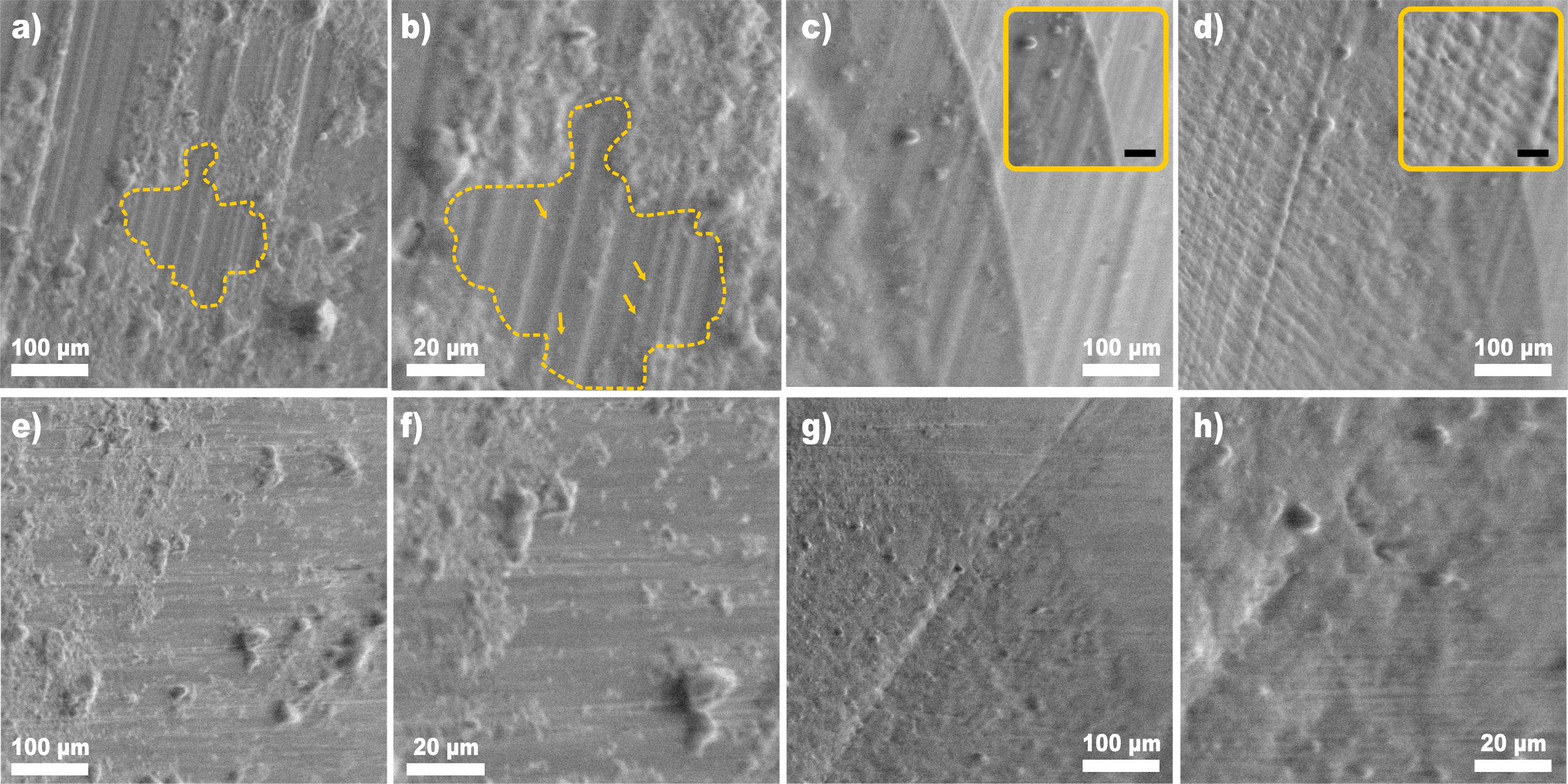


**Figure 4**: SEM imaging of amino-magnetic nanoparticles (NH_2_-MNP) as in Figure 1d and the final Poly-NH_2_-MNP compound as observed in Figure 1e. A drop of these samples was deposited on top of two different substrates of Al and Ti. The drops were dried in ambient conditions and further placed in ultra high vacuum conditions (UHV, ~10^-9^mbar). In a) and b) we can observe the NH_2_-MNP on aluminium, while e) and f) correspond to titanium. We confirmed the presence of MNP clusters from the NH_2_-MNP (yellow arrows) and the presence of a rough compact surface derived from the intrinsic magnetic interaction between the nanoparticles covered with APTES that may shrink during the drying process. In c) and d) we can observe the Poly-NH_2_-MNP compound on aluminium, while g) and h) correspond to titanium. We confirmed the formation of a smooth continuous morphology derived from the polymer coating. However small agglomeration sites can be evinced due to the intrinsic unaffected magnetic interaction between the nanoparticles. Insets scale bar 10 µm.


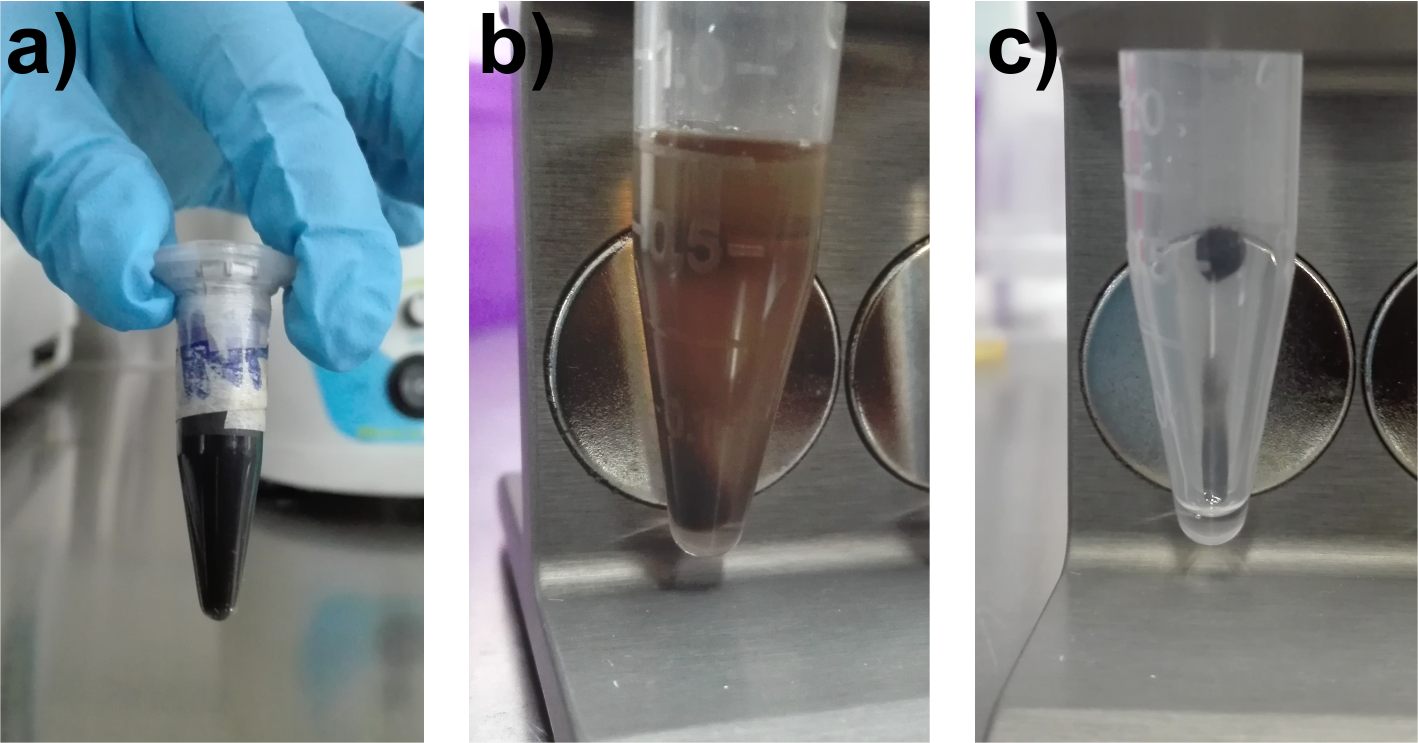


**Figure 5**: Poly-NH_2_-MNP compound used during the RNA extraction process. In a) the pristine Poly-NH_2_-MNP compound is observed. b) Poly-NH_2_-MNP compound during the first step of the magnetic extraction. The nanoparticles are strongly attracted to the magnet along the separation process protocol. c) final separation of the Poly-NH_2_-MNP compound after being washed and prepared for the final RT-PCT amplification analysis.


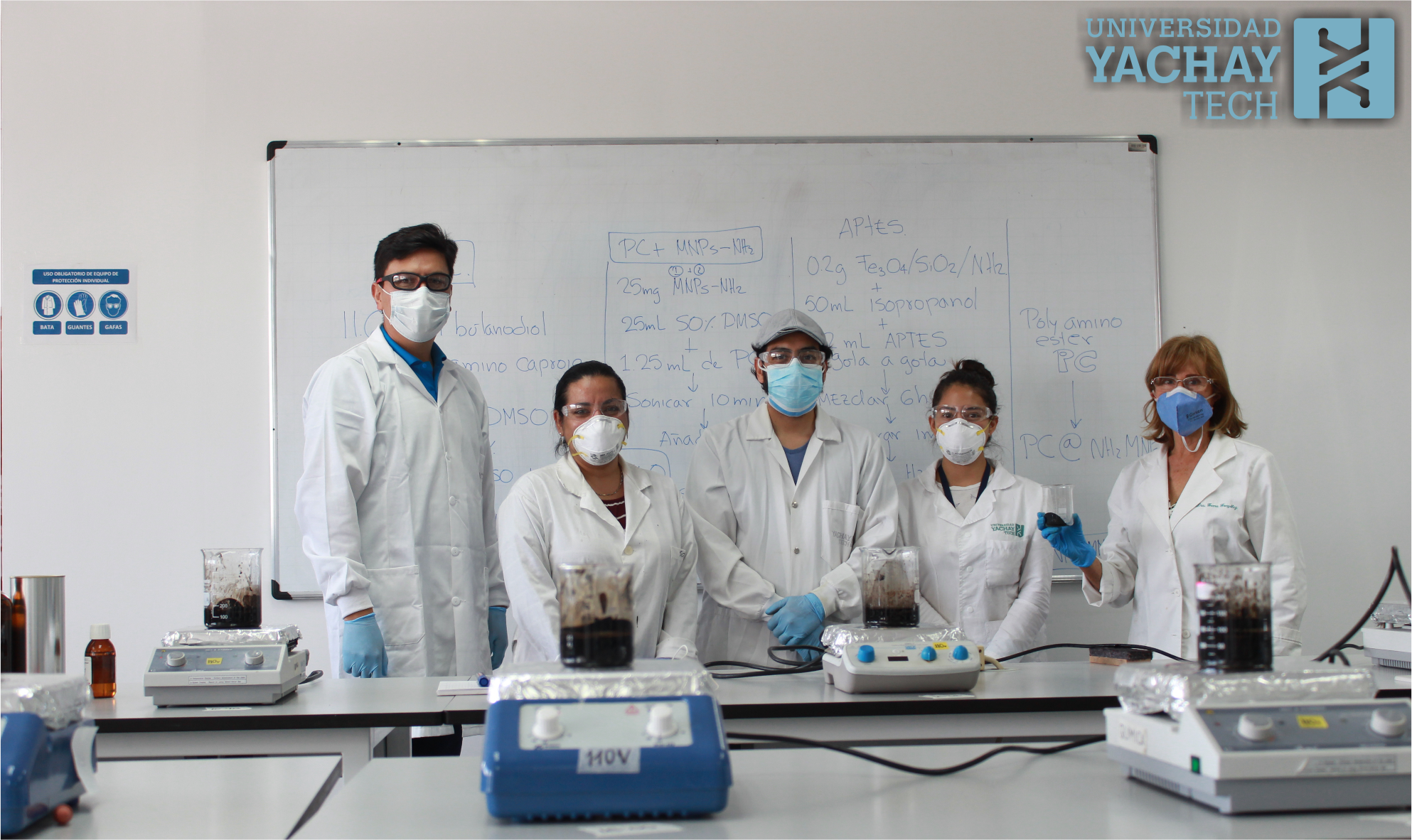


**Figure 6**: A photo of the team members who developed the experimental synthesis of magnetic nanoparticles for RNA extraction during the parallelization process for the MNP synthesis. From left to right: Dr. Carlos Reinoso, Dr. Sarah Briceño, Dr. Julio Chacón, Ing. Daniela Navas and Dr. Gema González.
